# Cost-effectiveness of a physical exercise programme for residents of care homes: a pilot study

**DOI:** 10.1186/s12877-016-0261-y

**Published:** 2016-04-18

**Authors:** Talitha I. Verhoef, Parita Doshi, Dan Lehner, Stephen Morris

**Affiliations:** Department of Applied Health Research, University College London, Gower Street, London, WC1E 6BT UK; Oomph! Wellness Ltd, London, UK

**Keywords:** Quality of life, Physical activity, Care homes, Cost-effectiveness

## Abstract

**Background:**

Oomph! Wellness organises interactive exercise and activity classes (Oomph! classes) for older people in care homes. We investigated the cost-effectiveness of Oomph! classes.

**Methods:**

Health-related quality of life was measured using the EQ-5D-5 L questionnaire at three time points; 3 months and 1 week prior to the start of the classes and after 3 months of Oomph! classes. Costs included the costs of organising the classes, training instructors and health service use (General Practitioner (GP) and hospital outpatient visits). To determine the cost-effectiveness of Oomph! classes, total costs and quality-adjusted life-years (QALYs) during the 3 months after initiation of the classes were compared to the total costs and QALYs of the 3 months prior to the classes and extrapolated to a 1-year time horizon. Uncertainty was taken into account using one-way and probabilistic sensitivity analysis.

**Results:**

Sixteen residents completed all three EQ-5D-5 L questionnaires. There was a decrease in mean health related quality of life per participant in the 3 months before Oomph! classes (0.56 to 0.52, *p* = 0.26) and an increase in the 3 months after the start of Oomph! classes (0.52 to 0.60, *p* = 0.06), but the changes were not statistically significant. There were more GP visits after the start of Oomph! classes and fewer hospital outpatient visits, leading to a slight decrease in NHS costs (mean £132 vs £141 per participant), but the differences were not statistically significant (*p* = 0.79). In the base case scenario, total costs for Oomph! classes were £113 higher per participant than without Oomph! classes (£677 vs £564) and total QALYs were 0.074 higher (0.594 vs 0.520). The incremental costs per QALY gained were therefore £1531. The 95 % confidence intervals around the cost/QALY gained varied from dominant to dominated, meaning there was large uncertainty around the cost-effectiveness results. Given a willingness to pay threshold of £20,000 per QALY gained, Oomph! classes had a 62 %–86 % probability of being cost-effective depending on the scenario used.

**Conclusions:**

Preliminary evidence suggests that Oomph! classes may be cost-effective, but further evidence is needed about its impact on health-related quality of life and health service use.

## Background

Only 36 % of men and 18 % of women aged 75 years or more in England get the minimum recommended levels of physical activity [1]. A recent study among older care home residents in the UK found that care home residents spent on average 79 % of their day as sedentary, 14 % in low, 6 % in light, and 1 % in moderate-to-vigorous physical activity [2]. In addition to low levels of physical activity, care home residents often have a lack of meaningful social interaction [3]. Regular exercise can improve physical function of frail older people [4]. If exercise training is combined with cognitive training, it can improve functional status as well as cognitive function in older adults with and without cognitive impairment [5]. Exercise—and wider meaningful activities—can also be a tool for building and cementing relationships, improving alertness and involvement, increasing confidence and creating a sense of wellbeing.

Oomph! (Our Organisation Makes People Happy) Wellness organises interactive exercise and activity classes (Oomph! classes) aimed at increasing the health and quality of life of older people in care homes. Classes are currently provided in several hundred care homes across the UK, focusing on chair-based exercise to music. Oomph! Wellness provides instructors to run classes, but also trains staff in care settings to run classes themselves. Oomph! classes are not a costless activity, and it is important to assess whether they represent value for money. The cost-effectiveness of several physical activity interventions in primary care or the community has been assessed [6], but the cost-effectiveness of such interventions in care homes have not been assessed. In this study, we used preliminary data to undertake a pilot study to investigate the cost-effectiveness of Oomph! classes, including their impact on health-related quality of life and health service use.

## Methods

We obtained data on Oomph! classes provided in 12 care homes throughout the UK between August 2014 and January 2015. Our pilot study aimed to involve 5 participants in each care home, 60 participants in total. The data were collected as part of a clinical audit of the Oomph! classes rather than as part of a research study. Approval by an ethics committee was therefore not required. Participants signed informed consent and were asked to complete 3 questionnaires over 6 months. All questionnaires were paper-based and were completed by residents with the assistance of the activities coordinator (staff employed by the care home) who subsequently collected the paperwork across all residents and posted this to the research team. The information from the questionnaires was used to assess the preliminary cost-effectiveness of the classes.

### Intervention

The content of the Oomph! classes vary from home to home, and day to day, because the classes are tailored to the tastes and abilities of individual residents. They were consistently chair-based, involving music, colourful props (e.g. pom poms and scarves) and story-telling imagery to accompany the exercise movements. Classes lasted from 20 min to 1 h (on average 40 min) and could have 3 to 20 people taking part in the same class. The number of sessions offered per week varied from home to home: the homes in the study offered between one to three sessions per week, which were run by activities coordinators (staff employed by the care home, trained by Oomph! Wellness to run Oomph! classes). Staff were trained to understand how to modify their exercises for people with different physical and mental health conditions and when rest or withdrawal from exercise might be appropriate.

### Participants

Participants in this study were all residents at homes owned by a private company that owns a portfolio of care homes across the UK. Oomph! classes are designed so that people of any age or ability can take part in them, unless they are absolutely bed-bound and immobile. The staff at the care homes identified residents to take part in the study based on their knowledge of previous participation levels in other activities. Staff were given strict instructions to select residents who had the capacity to give consent; therefore all residents provided their own consent. An information sheet was given and read out to residents so they fully understood what the study involved prior to giving consent. Limited data on participant characteristics such as age and gender were collected.

### Health-related quality of life

The effectiveness of the classes was assessed using information about the health-related quality of life of the participants, measured using the EQ-5D-5 L questionnaire [7] at three time points; 3 months prior to start of the classes, 1 week prior to the start of the classes, and after 3 months of Oomph! classes. The EQ-5D-5 L measures health using 5 levels of severity (no problem, slight problems, moderate problems, severe problems, extreme problems) in five dimensions of health-related quality of life (mobility, self-care, usual activities, pain/discomfort, and anxiety/depression). UK-specific value sets available from www.euroqol.org were used to translate responses into a utility score [8]. A score of 1 represents full health, a score of 0 represents death. Any value between 0 and 1 is possible, as well as negative values (representing a state worse than death) [8]. Because the EQ-5D-5 L instrument is completed at three points in the study it is possible to investigate the change in quality of life over time. All questionnaires were completed by the residents themselves. In some cases (e.g. poor eyesight or inability to write) the activities coordinator read out the question and wrote down the verbal response from the resident. For the analysis of health related quality of life, we only included participants who completed all three questionnaires.

### Costs

We included the costs of organising the classes, the costs of training instructors, and the costs of health service use among class attendees (General Practitioner (GP) and hospital outpatient visits). Costs were determined from the perspective of the NHS and presented in 2014 GBP. The number of GP visits and hospital outpatient visits for all participants was collected by the staff of the care homes for the 3 months prior to the classes and the 3 months following initiation of the classes. To analyse health service use we only included participants with complete data on health service use across the whole time period. Unit costs from routinely available sources [9] were applied to the number of visits to calculate the total costs of these visits for each participant. Unit costs for a GP visit were £46 and for a hospital outpatient visit they were £109 [9].

### Cost-effectiveness

Utility scores at the three time points (3 months prior to start of the classes, 1 week prior to the start of the classes and after 3 months of Oomph! classes) were used to calculate quality-adjusted life-years (QALYs) during the 3 months prior to the classes and the 3 months following initiation of the classes. Using extrapolation methods described below, we estimated total costs and QALYs per participant in 1 year with Oomph! classes (intervention group—based on the 3 months after start of the classes) and in 1 year without Oomph! classes (control group—based on the 3 months prior to the start of the classes). The total costs and QALYs in the intervention group (with Oomph! classes) were compared to the total costs and QALYs in the control group (without Oomph! classes). We do not have information about the quality of life and NHS costs after the first 3 months of participating in Oomph! classes; we therefore performed the cost-effectiveness analysis for three alternative scenarios (base case, best case and worst case, making different assumptions about costs and QALYs beyond the 3 month period (see below for further details). Incremental costs were divided by incremental QALYs to calculate the incremental cost-effectiveness ratio (the incremental cost per QALY gained).

### Sensitivity analysis

We accounted for uncertainty using one-way sensitivity analysis and probabilistic sensitivity analysis. In the one-way sensitivity analysis we varied each parameter one at a time over a plausible range (95 % confidence intervals were used for utilities and NHS costs, an extra wide range was used for costs of Oomph! classes per participant [£100 to £200]) and examined how the base case results changed. In the probabilistic sensitivity analysis, using random values from an appropriate distribution (reflecting the 95 % confidence intervals of each parameter), 1000 simulations were conducted. Based on these simulations, the probability that Oomph! classes would be cost-effective was calculated at different threshold values, along with 95 % confidence intervals around the incremental cost per quality-adjusted life-year (QALY) gained. When Oomph! classes were dominant, this means that QALYs were higher with Oomph! classes and costs were lower. When Oomph! classes were dominated, this means that QALYs were higher without Oomph! classes and costs were lower. Statistical analysis was undertaken using STATA version 13.1 and the economic modelling was undertaken using Microsoft Excel 2010.

## Results

In total, 102 surveys were collected from 48 residents from 9 care homes. We aimed to get all three questionnaires from each participant, but some only filled in one or two questionnaires. The first questionnaire (3 months Pre-Oomph! classes) was completed by 42 residents, the second (immediately Pre-Oomph! classes) by 30 residents and the last one (3 months Post-Oomph! classes) by 30 residents. In total, there were 16 residents (in 5 care homes) who completed all three EQ-5D-5 L questionnaires, and we used these for our base case analysis. All 16 residents had attended Oomph! classes. We did not have information on GP/hospital visits before and after Oomph! classes for all 16 participants who were included in the analysis of health related quality of life. Therefore data on NHS costs (calculated based on the number of GP/hospital visits) was based on a separate set of 17 residents for whom these data were available for the period before as well as after Oomph! classes (9 overlap with the set of 16 residents with complete quality of life data). Most respondents were female and most were 80–89 years of age (see Table 1).

**Table 1:** Gender and age distribution of respondents

	All respondents (*n* = 48)	Included for analysis on health related quality of life (*n* = 16)	Included for analysis on health service use (*n* = 17)
Gender			
Female	75.0 %	81.3 %	88.2 %
Male	6.3 %	6.3 %	-
Missing	18.8 %	12.5 %	11.8 %
Age			
70–79	14.5 %	18.8 %	17.6 %
80–89	41.7 %	43.8 %	47.1 %
90–99	25.0 %	25.0 %	29.4 %
>100	4.2 %	6.3 %	-
Missing	14.0 %	6.3 %	5.9 %

### Health related quality of life

The proportion of participants in the Oomph! classes reporting any problems in each domain of the EQ-5D-5 L questionnaire as well as utility scores at 3 months prior to intervention, 1 week prior to intervention and 3 months after intervention based on the 16 residents who completed all surveys are shown in Table 2. There were no statistically significant differences between the three time points (95 % confidence intervals overlap), but the point estimates indicate a decrease in health related quality of life in the 3 months before Oomph! classes (0.56 to 0.52, as might be expected in this population with increasing age) and an increase in the 3 months after the start of Oomph! classes (0.52 to 0.60).

**Table 2:** Results of the EQ-5D-5 L questionnaire (and 95 % confidence intervals) at the three different points in time

*n* = 16	3 months Pre-Oomph! classes	Immediately Pre-Oomph! classes	3 months Post-Oomph! classes
Proportion reporting any problems in domain			
Mobility	0.875 (0.57–0.97)	0.875 (0.57–0.97)	0.750 (0.46–0.91)
Self-care	0.625 (0.35–0.84)	0.813 (0.51–0.95)	0.750 (0.46–0.91)
Usual activities	0.563 (0.30–0.80)	0.500 (0.25–0.75)	0.375 (0.16–0.65)
Pain/discomfort	0.750 (0.46–0.91)	0.813 (0.51–0.95)	0.563 (0.30–0.80)
Anxiety/depression	0.625 (0.35–0.84)	0.563 (0.30–0.80)	0.438 (0.20–0.70)
Utility scores			
Unadjusted	0.562 (0.41–0.72)	0.520 (0.34–0.70)	0.604 (0.44–0.76)
Adjusted for Age and Gender	0.562 (0.40–0.73)	0.520 (0.36–0.68)	0.604 (0.46–0.75)

Full range of utility scores were -0.127 to 1 3 months pre-Oomph! classes, -0.142 to 1 Immediately Pre-Oomph! classes and -0.037 to 1 3 months post Oomph! classes

### Health service use

NHS costs among participants in the 3 months before and after the programme were based on the number of GP and hospital outpatient visits. Table 3 shows the number of GP visits, hospital outpatient visits and NHS costs before and after initiation of the programme. There were no statistically significant differences but the point estimates indicate more GP visits after the start of Oomph! classes and fewer hospital outpatient visits, leading to a slight decrease in NHS costs (mean value per participant £132 vs £141).

**Table 3:** Number of GP visits, hospital visits and NHS costs (and 95 % confidence intervals)

	Unadjusted		Adjusted for Age and Gender	
*n* = 17	During the 3 months before start of Oomph! classes	During the 3 months after start of Oomph! classes	During the 3 months before start of Oomph! classes	During the 3 months after start of Oomph! classes
GP visits	1.765 (1.04 ; 2.49)	2.235 (1.02 ; 3.45)	1.793 (1.06 ; 2.53)	2.201 (1.11 ; 3.29)
Hospital outpatient visits	0.529 (0.08 ; 0.98)	0.294 (−0.07 ; 0.66)	0.526 (0.12 ; 0.93)	0.296 (−0.05 ; 0.65)
NHS costs	£139 (£67 ; £211)	£135 (£57 ; £213)	£141 (£74 ; £209)	£132 (£67 ; £198)

### Costs of Oomph! classes

Costs of the Oomph! classes were calculated per home and per participant (Table 4). The costs of training activities coordinators including the time spent by the activities coordinator to do the training was £915. On average, each home organised 98.4 classes per year, in which the activities coordinator spent 50 min per class. Total costs for organising and delivering these classes were therefore £2555 per home per year. On average 17.2 residents per home participated in each classes (at least once per month), therefore the costs per participant were £148.55. The average number of classes per month and the average number of participants per home were calculated based on data from 159 care homes.

**Table 4:** Calculation of the costs of the Oomph! classes

Parameter name	Value	
Costs of training staff (activities coordinators) to run classes, per home	£915	Licence fee + 2 days staff time, incurred once
Number of classes per home	98.4 per year	Average of 8.2 per month in 159 care homes
Time spent per class	50 min	40 + 10 min preparation
Costs of activities coordinator	£20/hour	PPSRU—Agenda for Change band 2
Total costs per home per year	£2555	£915 + 98.4*50*20/60
Number of participants per home	17.2	Average number of participants (residents that attended at least one class per month) in 159 care homes
Total costs per participant per year ^a^	£148.55	2555/17.2

^a^ Total costs per participant are an average for all participants; some may only attend one class per month while others attend several classes per month

### Extrapolation of results to a 1-year time horizon

**Fig. 1:**
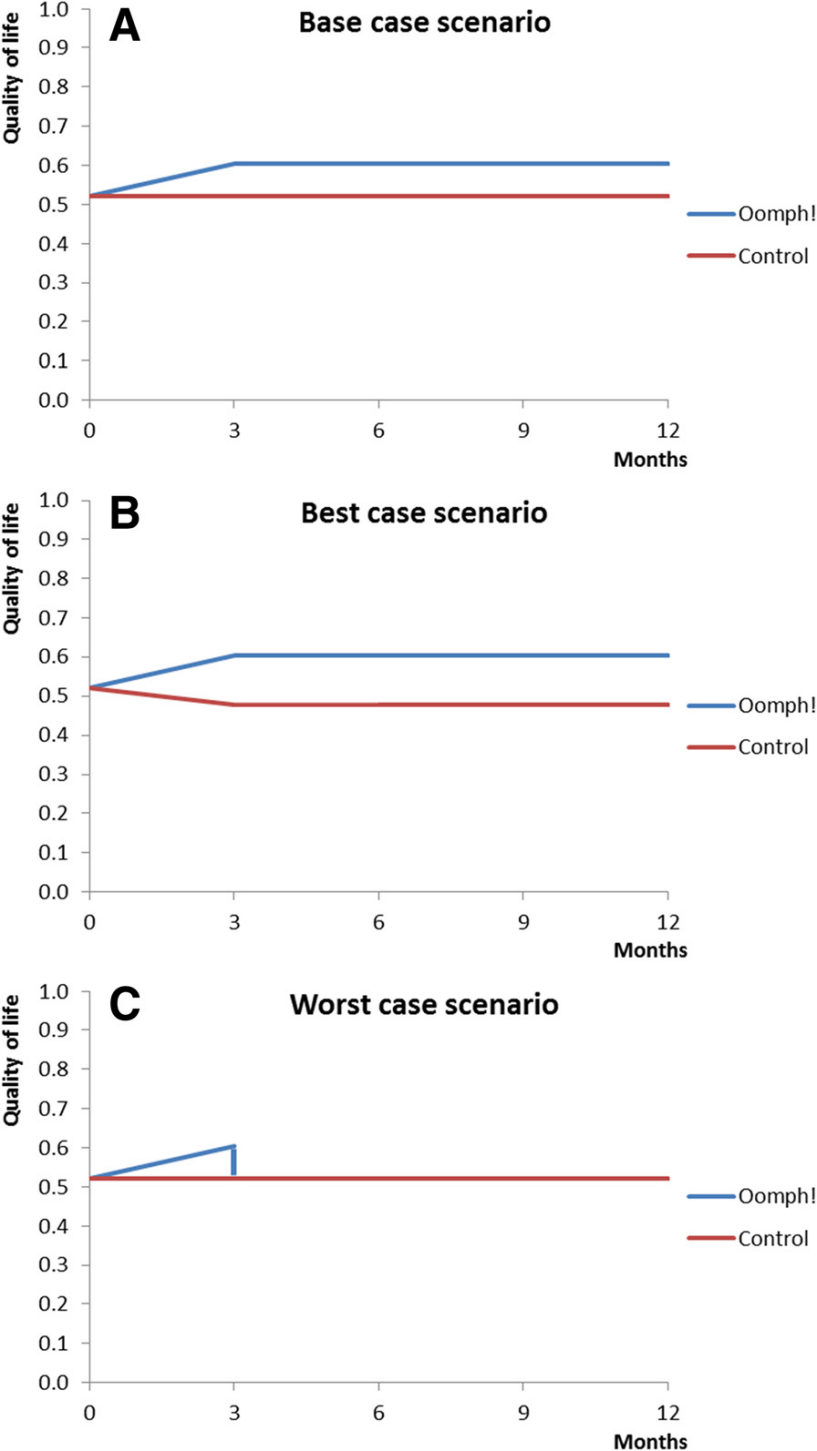
Alternative scenarios. These graphs show the different assumptions about the quality of life in the first year after the start of the Oomph! classes for each scenario. **a** Base case scenario, (**b**) Best case scenario, (**c**) Worst case scenario

Because we do not have information about the quality of life after the first 3 months of participating in Oomph! classes for the 16 residents who completed all questionnaires, we performed the analysis for three alternative scenarios (see Fig. 1). See Round et al [10] for an example of a study in a different participant group that extrapolates EQ-5D data in a similar way.

In the base case scenario, we assumed that in the absence of Oomph! classes (control group) health related quality of life would remain at 0.52 per participant during the entire year. The quality of life with Oomph! classes (intervention group) would increase in the first 3 months to 0.60 and remain at this level for the rest of the year (Fig. 1(a)). We assumed that the NHS costs would remain at the same level (£132 per participant in the intervention group and £141 in the control group per 3 months) for the whole year in this scenario. Because quality of life in the 3 months prior to Oomph! classes decreased by 0.042 we ran another scenario in which quality of life in the absence of Oomph! classes (control group) would not remain at 0.52 but would decrease by another 0.042 during months 3 to 6 and stay stable afterwards (Fig. 1(b)). This scenario was used as the best case scenario and used the same assumption about NHS costs as in the base case scenario. The least optimistic scenario (worst case scenario) was if there is only an increase in quality of life associated with Oomph! classes during the first 3months, but immediately after month 3 the quality of life returned to the same level as that seen in the absence of Oomph! classes (Fig. 1(c)). For the worst case scenario, we assumed that the costs in the intervention group would be £141 as in the control group from month 3 onwards.

### Cost-effectiveness

**Fig. 2:**
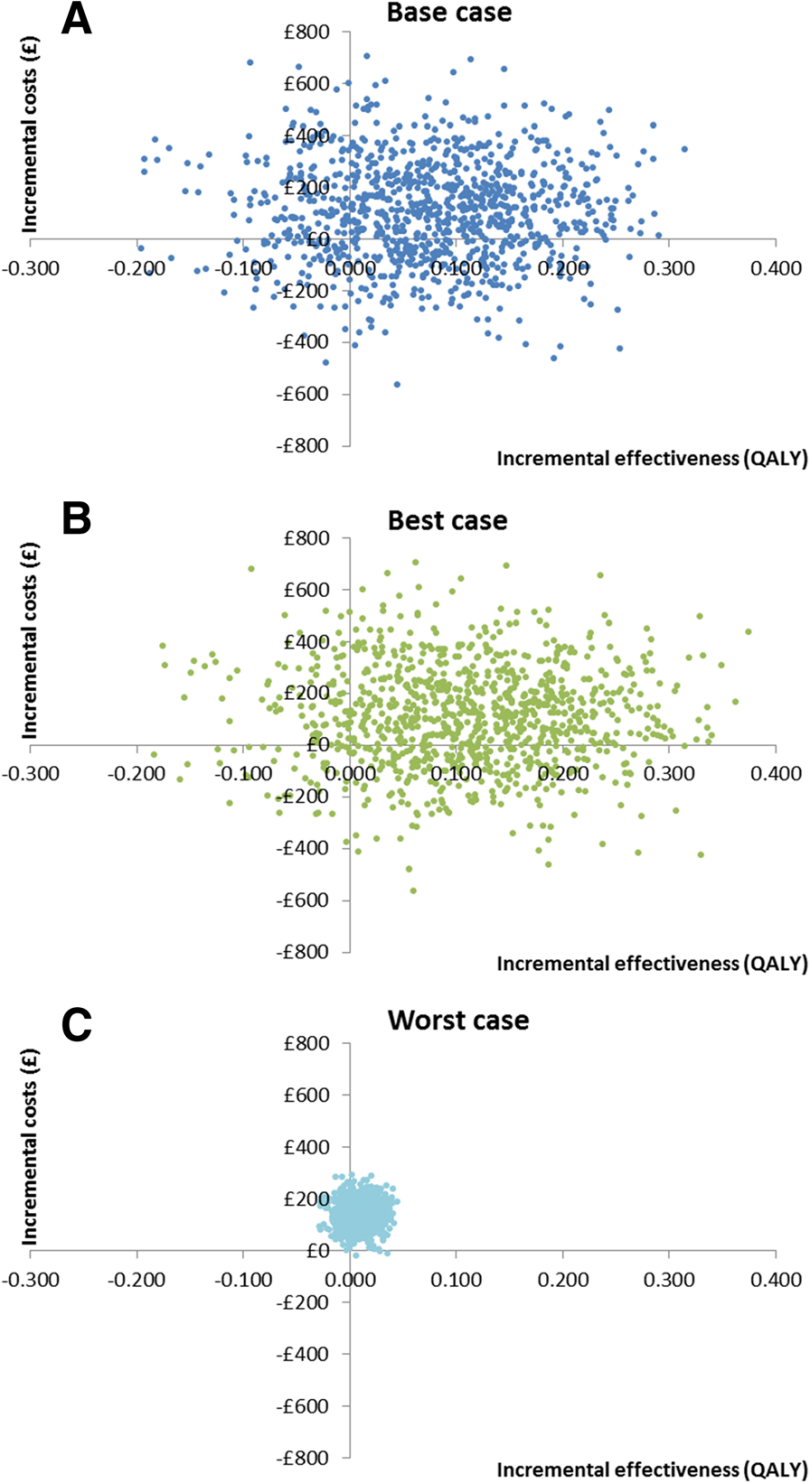
Scatter plot. These graphs show the incremental costs and incremental QALYs for each simulation of the probabilistic sensitivity analysis. **a** Base case scenario, (**b**) Best case scenario, (**c**) Worst case scenario

Incremental costs and QALYs gained were adjusted for age and gender. Total yearly costs per participant were based on 4 times the 3-monthly NHS costs in the control group and 4 times the 3-monthly NHS costs plus costs for Oomph! classes in the intervention group. In the base case scenario, 1-year costs were £113 higher (£677 vs. £564) with Oomph! classes than in the absence of Oomph! classes. QALYs increased by 0.074 (0.594 vs. 0.520) and the incremental costs per QALY gained were therefore £1531. In the best case scenario the incremental costs were the same as in the base case scenario, but incremental QALYs were 0.110 (0.594 vs. 0.483) and the incremental costs per QALY gained were £1021. In the worst case scenario, incremental costs were £140 (£704 vs. £564), incremental QALYs 0.011 (0.531 vs. 0.520) and the incremental costs per QALY gained £13,290. The 95 % confidence intervals around the incremental cost-effectiveness ratio varied from dominant to dominated (Table 5). The large confidence intervals mean there is substantial uncertainty around our cost-effectiveness results. This can also be seen on the scatter plot in Fig. 2, which shows the incremental costs and incremental QALYs for each simulation of the probabilistic sensitivity analysis.

**Table 5:** One-year costs and QALYs per participant and cost-effectiveness results for each scenario

Scenario		Costs (95 % confidence interval)	QALYs (95 % confidence interval)	Cost/QALY gained (95 % confidence interval)
Base case	Oomph! classes	£677 (443 to 974)	0.594 (0.468 to 0.702)	
	Control	£564 (331 to 867)	0.520 (0.373 to 0.666)	
	Increment	£113 (−265 to 496)	0.074 (−0.094 to 0.244)	£1531 ^a^ (Dominant [-£4931] to dominated [-£1604])
Best case	Oomph! classes	£676 (443 to 974)	0.594 (0.468 to 0.702)	
	Control	£564 (331 to 867)	0.483 (0.331 to 0.640)	
	Increment	£112 (−265 to 496)	0.110 (−0.077 to 0.290)	£1021 ^a^ (Dominant [-£4149] to dominated [-£2519])
Worst case	Oomph! classes	£704 (514 to 945)	0.531 (0.400 to 0.659)	
	Control	£564 (331 to 867)	0.520 (0.373 to 0.666)	
	Increment	£140 (42 to 237)	0.011 (−0.013 to 0.035)	£13,290 ^a^ (£2788 to dominated [-£7907])

^a^ £/QALY gained may differ from calculation of incremental costs/incremental QALYs as shown, due to rounding

### Sensitivity analysis

In the one-way sensitivity analysis we found that the total costs for the Oomph! classes per participant did not have a large influence on the costs per QALY gained. When these costs were varied from £100 to £200 per participant, the incremental costs per QALY gained varied from £871 to £2231 (Table 6). However, the NHS costs and health related quality of life with and without Oomph! classes did influence the results. When the NHS costs without Oomph! classes were varied over the 95 % confidence interval (£74 to £209), the costs per QALY gained varied from £5178 to a situation in which Oomph! classes were dominant. Oomph! classes were also dominant when the NHS costs with Oomph! classes were £67 (lower limit of 95 % confidence interval), but the costs per QALY gained increased to £5123 when these costs were £198 (upper limit of 95 % confidence interval). When the health related quality of life without Oomph! classes was varied from 0.36 to 0.68 the results varied from a costs per QALY gained of £527 to a situation in which Oomph! classes were dominated. Oomph! classes were also dominated when the health related quality of life with Oomph! classes was low (0.46) , but the costs per QALY gained decreased to £559 when this value was high (0.75).

**Table 6:** One-way sensitivity analysis

Scenario	Cost/QALY gained
Base case	£1531
Total costs for Oomph! classes per participant: £100	£871
Total costs for Oomph! classes per participant: £200	£2231
NHS costs per 3 months without Oomph! classes: £74	£5178
NHS costs per 3 months without Oomph! classes: £209	Oomph! classes are dominant
NHS costs per 3 months with Oomph! classes: £67	Oomph! classes are dominant
NHS costs per 3 months with Oomph! classes: £198	£5123
Utility immediately pre-Oomph! classes: 0.36	£527
Utility immediately pre-Oomph! classes: 0.68	Oomph! classes are dominated
Utility 3 months post-Oomph! classes: 0.46	Oomph!classes are dominated
Utility 3 months post-Oomph! classes: 0.75	£559

**Fig. 3:**
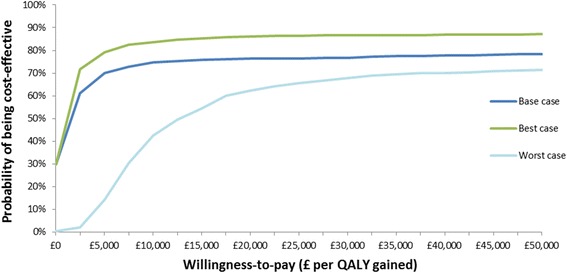
Cost-effectiveness acceptability curve. This graph shows for each scenario the probability that Oomph! classes would be cost-effective at different willingness to pay thresholds

In the probabilistic sensitivity analysis we found that given a willingness to pay threshold of £20,000 per QALY gained as recommended by the National Institute of Health and Care Excellence (NICE) [11], Oomph! classes have a 76 % probability of being cost-effective in the base case scenario. This probability was 86 % in the best case scenario and 62 % in the worst case scenario. Figure 3 shows a cost-effectiveness acceptability curve, portraying this probability for each scenario at different willingness to pay thresholds.

## Discussion

### Main findings

In our base case analysis, Oomph! classes increased QALYs in the first year after classes started, but also increased total costs. The incremental costs per QALY gained were £1531. In both our best and worst case scenario QALYs as well as costs were increased, and the point estimates of the incremental costs per QALY gained were below the willingness to pay threshold of £20,000 per QALY gained. However, there was considerable uncertainty around the effect of Oomph! classes on health related quality of life and NHS costs, which had a large influence on the cost-effectiveness estimates. Nevertheless, the probability that Oomph! classes would be cost-effective given a willingness to pay threshold of £20,000 per QALY gained was high (76 % in the base case scenario, 86 % in the best case scenario and even in the worst case scenario still 62 %).

### Comparison with other studies

To our knowledge, there are no published studies examining the cost-effectiveness of interactive exercise and activity classes aimed at improving quality of life of older people in care homes. In 2013, a study on exercise for depression in care home residents was published [12]. This study comprised a randomised controlled trial of a whole-home intervention including physiotherapist-led exercise. In the control group the prevalence of depression was lower and quality of life was higher than in the intervention group (which was more costly) and this intervention was therefore not cost-effective. A systematic review of the cost-effectiveness of physical activity interventions in primary care or the community showed that most of these interventions were cost-effective (costs per QALY gained were below £20,000 threshold for most interventions) [6]. However, interventions for which no direct supervision was required (such as walking or brief advice) had a higher chance of being cost-effective than supervised gym-based exercise or instructor-led programmes. In our study, the instructor-led Oomph! classes still seemed to be cost-effective based on the point estimates. Although some of the studies included in the review focussed on an elderly population, none of the studies assessed an intervention in care homes, such as the Oomph! classes.

There are several wellness initiatives of varying quality and penetration across the care home sector. There is little evidence of the cost-effectiveness of these initiatives and therefore it is difficult to compare them with Oomph! classes directly.

### Strengths and limitations

There are several limitations. The main limitation is the small sample size (only 16 for health related quality of life and 17 for NHS costs). The confidence intervals around the estimates for health related quality of life and NHS costs are therefore wide and we could not find any statistically significant differences in costs and outcomes. This subsequently causes large uncertainty around our cost-effectiveness estimates. Also, the sample used to measure changes in health related quality of life is not exactly the same as the sample used to measure GP visits and hospital outpatient visits (used to calculate NHS costs). There is some overlap, but we did not have information on GP/hospital visits before and after Oomph! classes for all 16 participants who were included in the analysis of health related quality of life, while for some participants not included in this analysis, we did have information on GP hospital visits before and after Oomph! classes. Ideally, the same sample would be used for both measures. Also, to calculate the costs of health service use we only collected data on hospital outpatient and GP visits. For a complete analysis of costs data on hospital inpatient stays, treatment by healthcare professionals other than the GP, ambulance use, and use of medication should be included as well. Based on the limited data in this study we expect that if there would be a difference in these costs between the two groups, it would favour Oomph! classes, but further research is needed. Study participants were selected by care home staff, and Oomph! classes were run by activities coordinators (staff employed by the care home) who also collected the data. There is therefore a risk of bias in the selection of participants and the data collection processes. Ideally the most appropriate participants would have been selected using strict inclusion and exclusion criteria that would not permit selection bias, and data would have been collected by independent researchers. We used a ‘before and after’ study design, therefore we could not separate the effect of Oomph! classes on health related quality of life from the effect of time (it might be expected that quality of life decreases over time in this elderly population). Also, we only measured quality of life and costs for 3 months after the first Oomph! classes and had to extrapolate these to a 1-year time horizon. The EQ-5D instrument has been criticised as not being sufficiently sensitive to detect meaningful changes in health–related quality of life, especially in people with milder conditions. This is thought to be a problem particularly with the 3-level version of the EQ-5D (EQ-5D-3 L). We used the new 5-level version of the EQ-5D (the EQ-5D-5 L), which is more sensitive to changes in health related quality of life [7]. We did not collect any information on medical comorbidities. This may have a large influence on health related quality of life and could also impact on the effect of the intervention.

### Further research

Because of the considerable uncertainty around the changes in health related quality of life and subsequently around the cost-effectiveness estimate, further research is necessary before a final conclusion about the cost-effectiveness of the Oomph! classes can be reached. This should be a comparative study with a larger sample size and longer follow-up than the current pilot study. In addition, future studies should collect data on medical comorbidities or impairments, so that the cost-effectiveness of the intervention can be assessed for different populations.

## Conclusion

Oomph! classes can improve the health and quality of life of older people in care homes in the short term and there is a large chance they would be cost-effective. There is however considerable uncertainty surrounding the cost-effectiveness estimates, and further research is needed.

### Ethics approval and consent to participate

The data were collected as part of a clinical audit of the Oomph! classes rather than as part of a research study. Approval by an ethics committee was therefore not required. Participants signed informed consent to participate.

### Consent for publication

Not applicable

### Availability of data and materials

The dataset supporting the conclusions of this article is included within the article.

## Abbreviations

GP: General Practitioner
NICE: National Institute of Health and Care Excellence
QALY: quality-adjusted life-year
